# Poly(A)-specific RNase (PARN) generates and regulates miR-125a-5p 3’-isoforms, displaying an altered expression in breast cancer

**DOI:** 10.1038/s41392-024-01795-3

**Published:** 2024-04-15

**Authors:** Luisa Tomasello, Shoshanah M. Holub, Giovanni Nigita, Rosario Distefano, Carlo M. Croce

**Affiliations:** 1 Department of Cancer Biology and Genetics, The Ohio State University, Columbus, OH USA; 2 Department of Internal Medicine, Division of Hematology, The Ohio State University, Columbus, OH USA

**Keywords:** Non-coding RNAs, Breast cancer

**Dear Editor**,

MicroRNAs (miRNAs) are small non-coding RNAs crucial for post-transcriptional gene regulation, processed from primary miRNA transcripts by Drosha and Dicer.^1^ Latest advancements in Next Generation Sequencing highlighted the existence of miRNA isoforms (isomiRs) resulting from alternative processing, RNA editing, or post-transcriptional modifications.^1^ IsomiRs can have distinct target preferences and diagnostic value in diseases like cancer.^1^ Exonucleases are enzymes cleaving RNA or DNA ends, playing a vital role in miRNA regulation.^2^ They control miRNA stability and biogenesis.^2^

We comprehensively profiled canonical and modified miRNAs in over 13,000 cancer samples across 38 distinct cohorts from The Cancer Genome Atlas Program (TCGA) and The Therapeutically Applicable Research to Generate Effective Treatments (TARGET).^1^ Nine miR-125a-5p 3’-isoforms appeared to be significantly dysregulated in 17 cancer types. An additional filtering retained miRNA isoforms having expression above the 25^th^ percentile of miR-125-5p 3’-isoforms in all comparisons in at least one condition (normal or tumor). Only four out of nine miRNA isoforms survived the filtering, specifically miR-125a-5p (0 | 0), miR-125a-5p (0 | -1), miR-125a-5p (0 | -2), and miR-125a-5p (0 | -3), with only miR-125a-5p (0 | 0), miR-125a-5p (0 | -2), and miR-125a-5p (0 | -3) being significantly dysregulated in at least 50% of the comparisons. Seven TCGA-cohorts presented all three isoforms significantly dysregulated in the tumor vs. normal comparison (Fig. 1a). Notably, in six out of seven cohorts, the expression of the two shorter isoforms (0 | -2) and (0 | -3) appeared downregulated in cancer, while the expression of the database-annotated molecule, miR-125a-5p (0 | 0), was higher in tumor samples (Fig. 1a). We investigated these isoforms in breast cancer (TCGA-BRCA), given that miR-125a-5p is considered a tumor suppressor in this type of cancer.^3^ Intending to elucidate whether these isoforms could have distinct roles, we initially confirmed through RNA-Immunoprecipitation analysis that argonaute RISC catalytic component 2 (AGO2) binds to all three molecules,^4^ thus suggesting that all three microRNA isoforms may potentially be functional. To determine whether they have antagonistic or supportive functions, we analyzed the genes dysregulated in patients belonging to the TCGA-BRCA cohort with the highest (75^th^ percentile) and the lowest expression (25^th^ percentile) of each miR-125a-5p isoform (Fig. 1b, left panel). The data obtained from this analysis were subsequently employed for an Ingenuity Pathway Analysis (IPA),^4^ which highlighted the involvement of the shorter isoforms of miR-125a-5p in the regulation of key cell cycle proteins.^4^

**Fig. 1:**
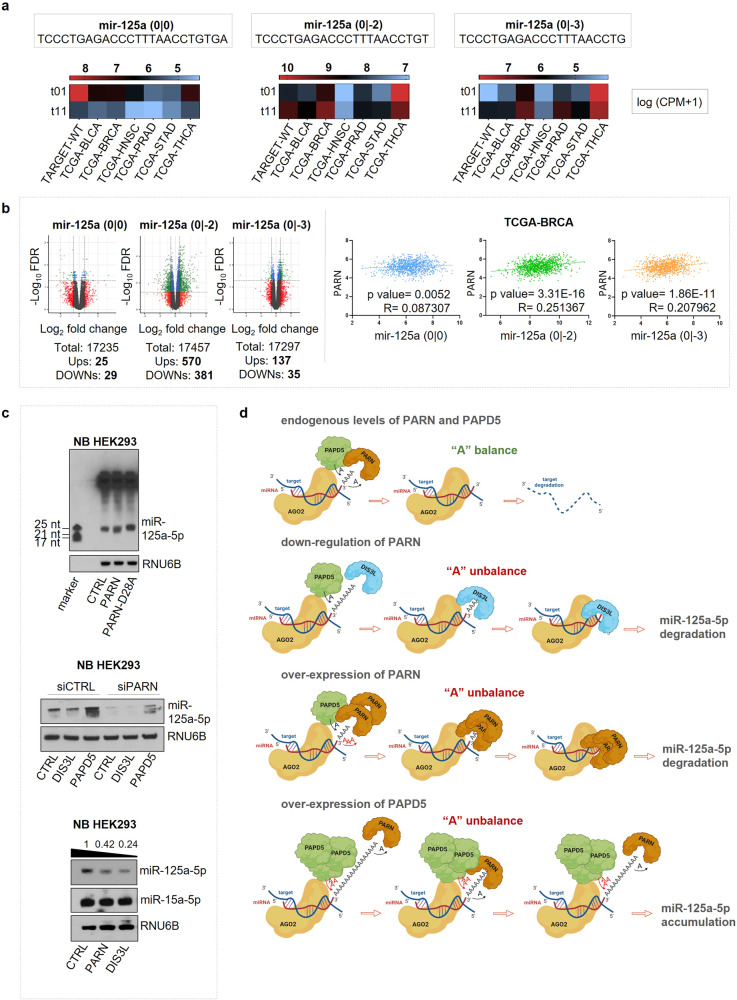
miR-125a-5p expression and regulation. **a** The heatmap illustrates the expression of miR-125a-5p isoforms across TCGA cohorts in log [Counts Per Million (CPM) + 1]. The Differential Expression (DE) analysis, made on cohorts with a minimum of five samples in at least one group (normal or tumor), demonstrated an up-regulation of miR-125a-5p (0 | 0) in primary solid tumor tissue (t01) compared with solid normal tissue (t11), and a lower expression of the two shorter isoforms in the t01 group. The isoforms selected in this study (geometric mean >3 RPM) were considered differentially expressed with |linear FC | >1.5 and FDR < 0.05. The data were normalized considering covariates such as tumor purity, pathologic stages, and age at initial pathologic diagnosis. **b** (left panel) Differentially expressed (DE) genes in Q1 and Q3 groups (respectively, 25^th^ and 75^th^ percentile of miRNA isoforms expression) of breast cancer patients (TCGA-BRCA cohort) with a |linear FC | >1.5 and FDR < 0.05. Gray dots represent NC, red dots represent log_2_ FC, blue dots represent FDR, and green dots represent FDR and log_2_ FC. (right panel) Correlation analysis between PARN and miR-125a-5p isoforms performed on TCGA-BRCA cohort patients showed a stronger positive correlation between PARN and miR-125a-5p (0 | -2) and miR-125a-5p (0 | -3) isoforms. Spearman correlation was applied to calculate significance and correlation coefficients. **c** (upper panel) Northern Blotting analysis demonstrated that upregulation of PARN wild-type in HEK293 cells leads to an evident shifting of miR-125a-5p, thus suggesting a partial degradation of this molecule that results in a shorter length of this microRNA. (middle and lower panels) Northern Blotting experiments show that the presence of PARN, PAPD5, and DIS3L strongly regulates the expression of miR-125a-5p. All the Northern Blotting were performed using TBE-Urea 15% polyacrylamide gels. A miRNA marker served as a reference for molecular weight. Loading RNA quantity and running were normalized using RNU6B as a control. The miR-15a-5p has been used as a control for the mechanism since it is a stable miRNA not affected by the action of exonucleases. **d** Graphic representation (Created with BioRender.com) of the molecular mechanism of miR-125a-5p 3’-isoforms regulation: the up or downregulation of PARN affects the expression of miR-125a-5p by altering the length of its poly(A) tail. For the same reason, the upregulation of PAPD5 also contributes to the accumulation of this molecule, preventing its excessive degradation by exonucleases like DIS3L

The presence of such a high number of 3’-end isoforms suggests that microRNA-125a-5p may undergo degradation mediated by a 3’-5’ exonuclease. The poly(A)-specific ribonuclease PARN is a 3’-5’ exonuclease responsible for trimming ~31% of cellular miRNAs.^5^ We have investigated the association between PARN and the three isoforms of miR-125a-5p in TCGA-BRCA patients, finding a stronger correlation between the exonuclease and the shorter isoforms (Fig. 1b, right panel). To confirm the role of this exonuclease in the generation of miR-125a-5p (0 | -2) and miR-125a-5p (0 | -3) isoforms, we transfected HEK293 cells with the wild-type PARN and the inactive mutant PARN-D28A. This mutant is catalytically inactive but still maintains the ability to bind substrates and is, therefore, expected to act as a trans-dominant negative mutant. To prevent excessive miRNA degradation resulting from exonuclease overexpression, we co-transfected the primiR-125a plasmid as well, thus restoring a stoichiometric balance.

The Northern Blotting analysis showed a shift in miR-125a-5p, attributed to up-regulated wild-type PARN protein compared to CTRL. The inactive mutant PARN-D28A, acting as a trans-dominant negative mutant, resulted in a slightly higher band than CTRL, reinforcing PARN’s involvement in miR-125a-5p trimming. (Fig. 1c, upper panel).

PARN not only controls the length of miR-125a-5p but also its stability. In fact, downregulating PARN results in a noticeable reduction of miR-125a-5p,^4^ possibly because the absence of PARN exposes this miRNA to become a target for more aggressive exonucleases.^2^

To understand the mechanisms underlying miR-125a-5p digestion in the absence of PARN, we examined endogenous miR-125a-5p expression following PARN-downregulation (siPARN) in combination with the overexpression of PAPD5 and DIS3L (DIS3-like exosome 3’-5’ exoribonuclease), a PARN-competitor exonuclease. To ensure that the downregulation of the microRNA in these experiments was due to post-transcriptional degradation, we treated cells with Actinomycin D for 6 h before the lysis to inhibit the transcription.^4^ We observed a general downregulation of miR-125a-5p following PARN-downregulation compared with the siCTRL (Fig. 1c, middle panel). However, there was a partial recovery of expression upon transfection with PAPD5, which increased miRNA expression even in the presence of PARN, thus suggesting that a longer poly(A)tail could have a shielding effect against 3’-5’ exonucleases. As expected, increased expression of DIS3L led to a pronounced degradation of miR-125a-5p, made even more evident by PARN downregulation (Fig. 1c, middle panel).

Despite PARN having a “protective” effect on miR-125a-5p against other exonucleases and the ability to work in tandem with PAPD5 to maintain the optimum length of the poly(A) tail, an excess of this protein (nearly 20 times higher than endogenous protein level)^4^ still leads to the 3’-degradation of the miRNA, albeit less efficiently than degradation mediated by DIS3L (Fig. 1c, lower panel). In fact, a mere 1.5-fold increase in the expression of this exonuclease^4^ is sufficient to achieve a twofold efficient degradation of miR-125a-5p (Fig. 1c, lower panel).

In summary, miR-125a-5p 3’ end isoforms appear to be regulated differently in normal and tumor tissues. Given the low number of genes dysregulated in the presence of high or low levels of canonical miR-125a-5p (0 | 0) in breast cancer patients (Fig. 1b), we hypothesize that this miRNA requires shortening to become functional. We have demonstrated that the exonuclease PARN contributes to the generation and the stability of miR-125a-5p 3’-isoforms, working in concert with PAPD5 in the addition and removal of untemplated A at the 3’ end, thereby preventing the rapid degradation of miR-125a-5p by antagonistic nucleases (DIS3L) with a similar substrate-affinity and a higher degradation efficiency (Fig. 1d).

It has been proved that alterations in the sequence or length of the microRNA 3’-end play a fundamental role in target recognition and regulatory efficiency, even if they do not directly affect the microRNA seed sequence.^1^ Considering the pivotal role that microRNAs have played over the years as significant diagnostic and prognostic cancer biomarkers, it now appears essential to shed light on the individual roles these isoforms play in the tumor onset and progression.

### Supplementary information

Supplementary Materials and Methods

## Data Availability

The source code of analyses and expression data generated in this study are available via the Zenodo repository (https://zenodo.org/record/6726643). Additional information, data, and tables revised by the reviewers but not included in this version of the manuscript are available via the Zenodo repository (https://zenodo.org/records/10794569).

## References

[1] Distefano R (2022). Pan-cancer analysis of canonical and modified miRNAs enhances the resolution of the functional miRNAome in cancer. Canc Res.

[2] Shukla S, Bjerke GA, Muhlrad D, Yi R, Parker R (2019). The RNase PARN controls the levels of specific miRNAs that contribute to p53 regulation. Mol. Cell.

[3] Guo X, Wu Y, Hartley R (2009). MicroRNA-125a represses cell growth by targeting HuR in breast cancer. RNA Biol..

[4] Tomasello, L., Holub, S. M., Nigita, G., Distefano, R. & Croce, C. M. Poly(A)-specific RNase (PARN) generates and regulates miR-125a-5p 3’-isoforms, displaying an altered expression in breast cancer (Supplementary Materials). (2024). 10.5281/ZENODO.10794568. 10.1038/s41392-024-01795-3 PMC11016533 38616203

[5] Lee D, Park D, Park JH, Kim JH, Shin C (2019). Poly(A)-specific ribonuclease sculpts the 3′ ends of microRNAs. RNA.

