# Supplementary material for: Poly(A)-specific RNase (PARN) generates and regulates miR-125a-5p 3’-isoforms, displaying an altered expression in breast cancer
Source: Signal Transduct Target Ther. 2024 Apr 15;9:90. doi: 10.1038/s41392-024-01795-3 (PMC11016533; doi:10.1038/s41392-024-01795-3)
Supplement: Supplementary file 1 — Supplementary Materials and Methods [file 41392_2024_1795_MOESM1_ESM.docx]

**Supplementary Materials for**

Poly(A)-specific RNase (PARN) generates and regulates miR-125a-5p 3’-isoforms, displaying an altered expression in breast cancer

Luisa Tomasello^1 †^, Shoshanah M. Holub^1^, Giovanni Nigita^1,*^, Rosario Distefano^2,*^, and Carlo M. Croce^1 †^

^1^Department of Cancer Biology and Genetics, The Ohio State University, Columbus, OH, USA.

^2^Department of Internal Medicine, Division of Hematology, The Ohio State University, Columbus, OH, USA.

^*^Equal contribution.

†Corresponding authors: luisa.tomasello@osumc.edu; [carlo.croce@osumc.edu](mailto:carlo.croce@osumc.edu).

**This PDF file includes:**

Supplementary materials and methods

**Supplementary Materials and Methods**

**Data sources and miRNA isoform annotation**

MiRNA-seq (v20) and Paired-end RNA-seq (v32) data were downloaded from 33 TCGA and 5 TARGET cohorts. Further details are available in our previous paper^1^.

**Cell lines**

HEK293 (ATCC; cat. #CRL-1573), were seeded and grown in RPMI-1641 medium (Millipore Sigma, CAT#: R8758) supplemented with 10% of FBS (Millipore Sigma, CAT#: F4135) and penicillin–streptomycin (100 U/mL penicillin and 0.1 mg/mL streptomycin; Millipore Sigma, CAT#: P0781). The authentication of the cell lines was executed through the short-tandem repeat profiling method. Mycoplasma test was regularly performed using the Mycoplasma PCR Detection Kit (Applied Biological Materials) and the MycoAlert ™ PLUS Mycoplasma Detection Kit (Lonza).

**Differential miRNA isoform expression analysis**

Differential expression (DE) analysis was performed considering only TCGA and TARGET cohorts with solid tissues characterized by at least five samples per group (e.g., primary tumor and normal tissues). At first, miRNA isoforms were filtered considering a minimum geometric mean expression >3 RPM in at least one of the two groups (e.g., tumor vs. normal). The resulting expression was further corrected for batch effects, considering standard covariates such as tumor purity and preclinical factors (e.g., gender, age at initial pathologic diagnosis, tumor stage, and platform). Finally, we retained molecules characterized by adjusted P-value <0.05 (Benjamini–Hochberg correction) and |linear fold change| >1.5. Further details are available in our previous paper^1^.

**Pathway enrichment analysis**

To investigate whether miR-125a-5p isoforms could play different roles in breast cancer, we separated TCGA-BRCA patients into two groups: patients with low (below 25^th^ quartile) and high (above 75^th^ quartile) expression of miR-125a-5p (0|0), miR-125a-5p (0|-2), miR-125a-5p (0|-3). Then, gene differential expression analysis was performed considering these two groups (for further information, see Distefano et al.^1^), retaining dysregulated genes with adjusted P-value <0.05 and |linear fold change| >1.5. Finally, dysregulated genes were employed to perform a pathway enrichment analysis using the Ingenuity® Pathway Analysis (IPA) software (v01-16). We considered only pathways with |z-score| ≥1.5 and p-value <0.01 (Supplementary Fig. 2a). For further information, see Distefano et al.^1^

**Cell lines transfection**

HEK293 cells were seeded at 75% of confluence in a 6-well plate two days before the transfection. We transfected 4 µg of the following plasmids: pCMV6-Entry Mammalian Expression Vector (Origene, CAT#: PS100001) as a control, PARN (NM_002582) Human Tagged ORF Clone (Origene, CAT#: RC207220), DIS3L (NM_133375) Human Tagged ORF Clone, (Origene, CAT#: RC201404), TRF4 2 (PAPD5) (NM_001040284) Human Tagged ORF Clone (Origene, CAT#: RC214986), and 100 nM of ON-TARGETplus Non-targeting Pool (Horizon Discovery, CAT#: D-001810-10-05), named siCTRL in this paper, and siGENOME Human PARN (5073) siRNA - SMARTpool (Horizon Discovery, CAT#: M-011348-00-005), named siPARN in this paper. The transfection was performed using 4 µL of Lipofectamine™ 2000 Transfection Reagent (Thermo Fisher Scientific, CAT#: 11668027) diluted in RPMI-1641 without FBS and antibiotics. After 5 hours, the transfection medium has been replaced with a complete medium. After 48-72 hours the cells were lysed for RNA or protein extraction.

**Correlation analysis**

Correlation analysis was performed leveraging the TCGA-BRCA expression data (solid primary tumor samples) generated in our previous work^1^. MiRNA isoforms and genes were initially filtered using a minimum geometric mean expression >3 RPM and >1 Transcripts Per Million (TPM), respectively, and then the expression was corrected for batch effects (tumor purity). Finally, the Spearman correlation was applied to calculate significance and correlation coefficients.

**Mutagenesis of PARN**

The plasmid PARN (NM_002582) Human Tagged ORF Clone (Origene, CAT#: RC207220) underwent a mutagenesis step to replace the 28th amino acid (Aspartate) with Alanine, thus affecting one of the four catalytic sites of this protein. PARN-D28A is an inactive 3'-5' exonuclease but it still retains the ability to bind the RNA. The mutagenesis was performed using QuickChange II XL Site-Directed Mutagenesis Kit (Agilent Technologies, CAT#:200521-5) following the manufacturer's instructions.

The mutagenesis primers were designed using the QuikChange Primer Design tool (agilent.com/store/primerDesignProgram.jsp):

**PARN_D28A_Fw** 5'-GACTTCTTCGCCATCGCTGGGGAGTTTTCAGGA-3'

**PARN_D28A_Rv** 5'-TCCTGAAAACTCCCCAGCGATGGCGAAGAAGTC-3'

The mutated plasmid was subsequently subjected to Sanger sequencing to verify mutagenesis.

**Northern Blotting**

25 µg of RNA isolated from cells and quantified as described above (See section “RNA isolation, reverse transcription, and real-time RT-PCR”) were mixed 1:1 with NOVEX® TBE-Urea Sample Buffer (2X) (Thermo Fisher Scientific, CAT#: LC6876) and denatured at 70°C for 10 minutes. Then, denatured RNAs were loaded onto 15% TBE-Urea Gels (Thermo Fisher Scientific, CAT#: EC6885BOX) and subject to electrophoresis at 150 V, in TBE 1X (National Diagnostic), for about two hours. A microRNA Marker (New England BioLabs, CAT#: N2102S) was used according to the manufacturer`s instructions to assess the molecular weight of the miR-125a-5p isoforms. After the run, the gel was stained with Ethidium Bromide (Bio-Rad), diluted at the concentration of 0.5 µg/mL in TBE 1X, to check the RNA quality and then subject to electroblotting. The samples were transferred to a positive charged nylon membrane, Hybond™ -N^+^ (GE Healthcare) in cold TBE 1X, at 360 mA, for three hours. After the transfer, the membrane underwent crosslinking of RNA through ultraviolet light and then has been incubated with ULTRAhyb™ Ultrasensitive Hybridization Buffer (Thermo Fisher Scientific, CAT#: AM8670) at 42°C, with gentle rocking for 30 minutes. Then, 5’ dual biotin-labeled probes (Integrated DNA Technologies), complementary to miR-125a-5p, miR-15a-5p, RNU6B, and microRNA marker (See sequences listed below), were added to the Hybridization buffer with a final concentration of 10 nM for microRNAs and RNU6B probes and 0.1 nM for the microRNA marker probe. After two O/N incubations with probes, the signal was detected using Chemioluminescent Nucleic Acid Detection Module (Thermo Fisher Scientific, CAT#: 89880), according to the manufacturer`s instructions.

Northern Blotting probes:

**miR-125a-5p**: 5’- /52-bio/ AGGTTAAAGGGTCTCAGGGA - 3’

**miR-15a-5p**: 5’- /52-bio/ CAAACCATTATGTGCTGCTA - 3’

**RNU6B (control)**: 5’- /52-bio/ GTGCTGCCGAAGCGAGCAC- 3’

**microRNA marker**: 5’- /52-bio/ AGGTTAAAGGGTCTCAGGGA- 3’

**References of Supplementary Materials**

1. Distefano, R. *et al.* Pan-Cancer Analysis of Canonical and Modified miRNAs Enhances the Resolution of the Functional miRNAome in Cancer. *Canc Res* **82**, 3687–3700 (2022).
